# Supplementary material for: Impact of malaria diagnostic choice on monitoring of Plasmodium falciparum prevalence estimates in the Democratic Republic of the Congo and relevance to control programs in high-burden countries
Source: PLOS Glob Public Health. 2023 Jul 26;3(7):e0001375. doi: 10.1371/journal.pgph.0001375 (PMC10370698; doi:10.1371/journal.pgph.0001375)
Supplement: S1 Table — (DOCX) [file pgph.0001375.s001.docx]

**S1 Table. Primer sequences and reaction conditions** for the real-time PCR assay targeting the *P.* *falciparum*-specific lactate dehydrogenase (*pfldh*) gene*.*

|  | ***P. falciparum-*specific *pfldh*** | | | | | |
| --- | --- | --- | --- | --- | --- | --- |
| *Adapted from:* | Pickard AL et al. Antimicrob Agents Chemo 2003. 47(8):2418-2423.[1] | | | | | |
| *Forward Primer (5'->3')* | ACGATTTGGCTGGAGCAGAT | | | | | |
| *Reverse Primer (5'->3')* | TCTCTATTCCATTCTTTGTCACTCTTTC | | | | | |
| *Probe (5'->3')* | FAM/ AGTAATAGTAACAGCTGGATTTACCAAGGCCCCA /TAMRA | | | | | |
| *Cycling conditions:* | Temp | | | Duration | No. Cycles |  |
|  | 50C | | | 2 min | x1 |  |
|  | 95C | | | 10 min | x1 |  |
|  | 95C | | | 15 sec | x40 |  |
|  | 60C | | | 1 min |  |  |
| *Reaction conditions:* | Roche FastStart Universal Probe Master (Rox) | | | | | |
|  | Fwd primer | 200nM |  | | | |
|  | Rev primer | 200nM |  | | | |
|  | Probe | 100nM |  | | | |
|  | Template | 2 µl |  | | | |
|  | Total volume | 12 µl |  | | | |
