# Supplementary material for: Impact of malaria diagnostic choice on monitoring of Plasmodium falciparum prevalence estimates in the Democratic Republic of the Congo and relevance to control programs in high-burden countries
Source: PLOS Glob Public Health. 2023 Jul 26;3(7):e0001375. doi: 10.1371/journal.pgph.0001375 (PMC10370698; doi:10.1371/journal.pgph.0001375)
Supplement: S2 Table — (DOCX) [file pgph.0001375.s002.docx]

**S2 Table. Positive and negative predictive values of malaria diagnostic methods versus PCR and BBA for the detection of *Plasmodium* *falciparum*, stratified by health area and malaria prevalence.**

| **Health area**  *prevalence* | **Diagnostic test** | **Versus PCR** | | **Versus BBA** | |
| --- | --- | --- | --- | --- | --- |
|  |  | **PPV**  **(95% CI)** | **NPV**  **(95% CI)** | **PPV**  **(95% CI)** | **NPV**  **(95% CI)** |
| Bu  *high* | RDT | 84.6 (79.9–88.7) | 58.8 (51.8–65.5) | 96.1 (93.1–98.0) | 59.7 (52.8–66.4) |
|  | Micro | 86.0 (81.6–89.8) | 64.0 (56.8–70.7) | 88.4 (84.2–91.9) | 52.3 (45.1–59.4) |
|  | PCR | N/A | N/A | 87.3 (83.2–90.8) | 57.5 (49.6–65.1) |
|  | BBA | 79.9 (75.4–84.0) | 70.1 (61.7–77.6) | N/A | N/A |
| Kimpoko  *moderate* | RDT | 93.6 (88.8–96.8) | 66.2 (61.0–71.1) | 86.0 (80.0–90.8) | 65.3 (60.1–70.3) |
|  | Micro | 76.8 (71.2–81.8) | 70.2 (64.2–75.7) | 67.3 (61.3–72.9) | 64.3 (58.2–70.2) |
|  | PCR | N/A | N/A | 67.3 (61.3–72.9) | 64.3 (58.2–70.2) |
|  | BBA | 75.5 (69.9–80.5) | 69.8 (63.8–75.4) | N/A | N/A |
| Voix du Peuple  *low* | RDT | 57.1 (28.9–82.3) | 95.8 (93.4–97.5) | 85.7 (57.2–98.2) | 94.3 (91.6–96.4) |
|  | Micro | 32.4 (17.4–50.5) | 96.4 (94.0–98.0) | 38.2 (22.2–56.4) | 94.3 (91.5–96.4) |
|  | PCR | N/A | N/A | 52.0 (31.3–72.2) | 94.4 (91.7–96.5) |
|  | BBA | 37.1 (21.5–55.1) | 96.9 (94.6–98.4) | N/A | N/A |
| All | RDT | 87.1 (83.7–90.0) | 77.0 (74.2–79.6) | 92.1 (89.2–94.3) | 76.3 (73.5–78.9) |
|  | Micro | 78.8 (75.3–82.1) | 80.7 (77.9–83.3) | 76.1 (72.5–79.5) | 75.2 (72.2–78.1) |
|  | PCR | N/A | N/A | 79.5 (76.1–82.6) | 80.2 (77.3–82.9) |
|  | BBA | 75.8 (72.4–79.1) | 83.3 (80.5–85.9) | N/A | N/A |

*Abbreviations*: 95% CI, 95% confidence interval; BBA, bead-based immunoassay; Micro, microscopy; N/A, not applicable; NPV, negative predictive value, PCR, polymerase chain reaction; PPV, positive predictive value; RDT, rapid diagnostic test.
