# Supplementary material for: Impact of malaria diagnostic choice on monitoring of Plasmodium falciparum prevalence estimates in the Democratic Republic of the Congo and relevance to control programs in high-burden countries
Source: PLOS Glob Public Health. 2023 Jul 26;3(7):e0001375. doi: 10.1371/journal.pgph.0001375 (PMC10370698; doi:10.1371/journal.pgph.0001375)
Supplement: S1 Text — (DOCX) [file pgph.0001375.s003.docx]

**S1 Text. Detailed methods used to estimate the number of *Plasmodium falciparum* infections in Kinshasa Province** in March-June 2018 by malaria diagnostic method, derived from the Democratic Republic of the Congo (DRC) Ministry of Health age distribution and population estimates (8,993,453 people).

*Purpose:* To inform the potential impact of the choice in malaria diagnostic method on malaria control programs – for example, during decision making about rapid diagnostic test or antimalarial medication procurement – we estimated the number of individuals with *P. falciparum* malaria infection in Kinshasa Province during the baseline data collection period (March-June 2018) using each diagnostic method: RDT, microscopy, *pfldh* real-time polymerase chain reaction (PCR), and multiplex bead-based immunoassay. To calculate and compare *P. falciparum* prevalence by diagnostic assay, we used publicly available DRC health area shapefiles (boundaries) and age-stratified population estimates obtained from the DRC Ministry of Health for 2018 and derived from the DRC District health Information System 2 (DHIS2).

*Step 1: Estimate P. falciparum infection prevalence by health area in Kinshasa province using DRC Demographic and Health Survey (2013-2014)*

The 2013-2014 Demographic and Health Survey (DHS) is a nationally representative, cross-sectional survey that collected dried blood spots (DBS) from 17,765 adults (>16 years old) and 8,696 children (<5 years old) across 535 clusters, which are village locations with spatial information. These DBS were assessed for *P. falciparum* infection at the University of North Carolina at Chapel Hill using *pfldh* PCR, as has been described previously [6]. To assign the health areas in Kinshasa province to high-, medium-, and low-prevalence categories, we first calculated the cluster-level malaria prevalence based on all samples for each DHS cluster. Next, we fit a Thin Plate Spline model to the cluster-level prevalence values to spatially interpolate the malaria prevalence across Kinshasa province in areas without DHS clusters [7]. Then, we downloaded DRC health area shapefiles (boundaries) updated on 28 June 2022 from the Geo-Referenced Infrastructure and Demographic Data for Development 3 (GRID3) website [8]. Among the 416 health areas in Kinshasa province according to Ministry of Health population data derived from DHIS2, we identified 404 (97.1%) health area boundaries. We were able to match 402 (96.6%) health areas in the health boundaries file with the population data by name. Finally, we used zonal statistics in to estimate the mean *P. falciparum* infection prevalence in each of the 402 health area boundaries within Kinshasa province.

*Step 2: Stratify health areas in Kinshasa Province into three malaria prevalence categories*

We stratified health areas in Kinshasa Province into three malaria prevalence categories. If they had a prevalence of 15-24% (low), 25-34% (moderate), or ≥35% (high) using the PCR-based prevalence estimates from the 2013-2014 DHS survey described above. We chose these thresholds because each contained one of the study health areas: Voix du Peuple (17.0%), Kimpoko (30.4%), and Bu (40.5%) – **see Fig 3** in manuscript. Next, we matched each health area in Kinshasa Province with one of the study health areas.

*Step 3: Estimate age-specific malaria prevalence estimates from the study health areas.*

We estimated age-specific malaria prevalence estimates by RDT, microscopy, PCR, and bead-based immunoassay from the study health areas (**Table A**). For this analysis, we stratified age into two categories, <5 and ≥5 years old using age data used and provided by the DRC Ministry of Health. Fourth, we generated age-specific weights by multiplying the study age-specific prevalence estimates and age distribution (<5 years: 18.9%; ≥5 years: 81.1%) used by the DRC Ministry of Health.

**Table A. Age-specific (<5 and ≥ 5 years) malaria prevalence and 95% confidence interval by malaria diagnostic method (RDT, Microscopy, PCR and Luminex).**

| **Area** | **Study Population**  **N (%)** | **Prevalence (95% CI)** | | | |
| --- | --- | --- | --- | --- | --- |
|  |  | **RDT^a^** | **Microscopy^b^** | **PCR^c^** | **BBA^d^** |
| Bu |  |  |  |  |  |
| <5 | 119 (21.2) | 60.4 (50.2-69.8) | 53.3 (43.1-63.3) | 50.3 (40.0-60.5) | 72.7 (62.5-81.0) |
| ≥ 5 | 442 (78.8) | 56.7 (50.8-62.4) | 61.2 (55.1-66.9) | 70.2 (63.6-76.0) | 72.4 (66.4-77.7) |
| Kimpoko |  |  |  |  |  |
| <5 | 77 (13.6) | 16.2 (8.7-28.2) | 38.4 (27.6-50.4) | 29.6 (19.7-41.8) | 32.2 (22.1-44.4) |
| ≥ 5 | 491 (86.4) | 35.0 (30.2-40.1) | 52.4 (47.1-57.7) | 56.3 (50.8-61.6) | 53.6 (48.1-58.9) |
| Voix du Peuple |  |  |  |  |  |
| <5 | 38 (9.0) | 0.0 (0.0- 0.0) | 16.1 (6.2-35.8) | 2.0 (0.2-14.4) | 0.0 (0.0- 0.0) |
| ≥ 5 | 382 (91.0) | 3.7 (1.8- 7.4) | 7.1 (4.9-10.3) | 6.1 (3.8- 9.5) | 9.1 (9.1- 9.1) |

*Abbreviations*: BBA, bead-based immunoassay; PCR, polymerase chain reaction; RDT, rapid diagnostic test.

^a^ HRP2-band positive, ^b^ Any *Plasmodium* species visualized, ^c^ *P. falciparum* lactate dehydrogenase PCR-positive, ^d^ *P. falciparum* HRP2 antigen positive

*Step 4: Estimate the number of Plasmodium falciparum infections in Kinshasa Province in in March-April 2018 by diagnostic assay*

We multiplied the age-specific weights by the age-stratified population estimates obtained from the DRC ministry of health for 2018 to obtain an age-specific number of individuals with malaria by diagnostic method. Finally, we summed the estimated number of cases by diagnostic method (**Table 3** in manuscript).

**References**

1.    Pickard AL, Wongsrichanalai C, Purfield A, Kamwendo D, Emery K, Zalewski C, et al. Resistance to antimalarials in Southeast Asia and genetic polymorphisms in pfmdr1. Antimicrob Agents Chemother. 2003;47: 2418–2423. doi:10.1128/AAC.47.8.2418-2423.2003

2.    Hofmann N, Mwingira F, Shekalaghe S, Robinson LJ, Mueller I, Felger I. Ultra-sensitive detection of Plasmodium falciparum by amplification of multi-copy subtelomeric targets. PLoS Med. 2015;12: e1001788. doi:10.1371/journal.pmed.1001788

3.    Veron V, Simon S, Carme B. Multiplex real-time PCR detection of P. falciparum, P. vivax and P. malariae in human blood samples. Exp Parasitol. 2009;121: 346–351. doi:10.1016/j.exppara.2008.12.012

4.    Srisutham S, Saralamba N, Malleret B, Rénia L, Dondorp AM, Imwong M. Four human Plasmodium species quantification using droplet digital PCR. PLoS ONE. 2017;12: e0175771. doi:10.1371/journal.pone.0175771

5.    Perandin F, Manca N, Calderaro A, Piccolo G, Galati L, Ricci L, et al. Development of a real-time PCR assay for detection of Plasmodium falciparum, Plasmodium vivax, and Plasmodium ovale for routine clinical diagnosis. J Clin Microbiol. 2004;42: 1214–1219. doi:10.1128/JCM.42.3.1214-1219.2004

6.    Deutsch-Feldman M, Aydemir O, Carrel M, Brazeau NF, Bhatt S, Bailey JA, et al. The changing landscape of Plasmodium falciparum drug resistance in the Democratic Republic of Congo. BMC Infect Dis. 2019;19: 872. doi:10.1186/s12879-019-4523-0

7.    Nychka D, Furrer R, Paige J, Sain S. fields: Tools for spatial data. N/A; 2021.

8.    Geo-Referenced Infrastructure and Demographic Data for Development 3 (GRID3). GRID3 DRC Health Area Boundaries - Haut-Katanga, Kasaï, Kasaï-Oriental, Kinshasa, and Lomami Provinces, Version 01. In: GRID3 DATA HUB [Internet]. [cited 22 Oct 2022]. Available: https://data.grid3.org/datasets/GRID3::grid3-drc-health-area-boundaries-haut-katanga-kasa%C3%AF-kasa%C3%AF-oriental-kinshasa-and-lomami-provinces-version-01/about
