# Supplementary material for: Impact of malaria diagnostic choice on monitoring of Plasmodium falciparum prevalence estimates in the Democratic Republic of the Congo and relevance to control programs in high-burden countries
Source: PLOS Glob Public Health. 2023 Jul 26;3(7):e0001375. doi: 10.1371/journal.pgph.0001375 (PMC10370698; doi:10.1371/journal.pgph.0001375)
Supplement: Attachment — Submitted filename: Response to Reviewers_clean.pdf [file pgph.0001375.s004.pdf]

PLoS Global Public Health
1265 Battery Street
Suite 200
San Francisco, CA 94111
USA

February 24, 2023

Re: PGPH-D-22-01821

Dear Dr. Srinivasa Rao Mutheneni,

Thank you and the reviewers for your positive review of our manuscript. On behalf of my co-authors, I am happy to submit this revision for further consideration. Below we provide a point-by-point response to the reviewers' and editor's suggestions.

We look forward to your review.

Sincerely,

Jonathan B. Parr, MD, MPH
Assistant Professor of Medicine
Division of Infectious Diseases

The University of North Carolina School of Medicine, Division of Infectious Diseases
130 Mason Farm Rd., Chapel Hill, NC 27599 | jonathan_parr@med.unc.edu | 1-919-843-4132 | ideelresearch.org

Journal Requirements:

Journal requirements 1: Please send a completed 'Competing Interests' statement, including any COIs declared by your co-authors. If you have no competing interests to declare, please state "The authors have declared that no competing interests exist". Otherwise please declare all competing interests beginning with the statement "I have read the journal's policy and the authors of this manuscript have the following competing interests:"

> We have added the statement to the 'Competing Interest' section: All other authors have declared that no competing interests exist.

Journal requirement 2a: State the initials, alongside each funding source, of each author to receive each grant.

> This information is included in the Funding section of the grant.

Journal requirement 2b: State what role the funders took in the study. If the funders had no role in your study, please state: "The funders had no role in study design, data collection and analysis, decision to publish, or preparation of the manuscript."

> The funders had no role in the study. We have added this sentence as suggested.

Journal requirement 2c: If any authors received a salary from any of your funders, please state which authors and which funders.

> Authors received funding from their home institutions, which received financial support from these grants. None of the authors received salary directly from the funders.

Journal requirement 3: In the online submission form, you indicated that your data will be submitted to a repository upon acceptance. We strongly recommend all authors deposit their data before acceptance, as the process can be lengthy and hold up publication timelines. Please note that, though access restrictions are acceptable now, your entire data will need to be made freely accessible if your manuscript is accepted for publication. This policy applies to all data except where public deposition would breach compliance with the protocol approved by your research ethics board. If you are unable to adhere to our open data policy, please kindly revise your statement to explain your reasoning and we will seek the editor's input on an exemption. Please be assured that, once you have provided your new statement, the assessment of your exemption will not hold up the peer review process.

> **Data availability:** De-identified data is available through the Carolina Digital Repository (https://cdr.lib.unc.edu/concern/data_sets/0c483v220?locale=en).

Response to Editors and Reviewers

Reviewer 1: Thank you for the submission. This is a research with a well-constructed method and execution. Its findings resonate to medical practice not only in low- and middle-income countries but all over the world where travel medicine is practiced. The limitations are also well-outlined.

Thank you for your time and positive review of our manuscript.

Reviewer 2, Comment 1: Specify on Methods that samples used in this analysis were collected in March-June 2018, as indicated in the Abstract.

Thank you for the suggestion. The information was already in the first paragraph the methods in the original submission.

Reviewer 2, Comment 2: I suggest to add how many individuals had available results for only some of the diagnostic assays. Were these individuals different than the 1431 individuals finally included?

Among the 1,450 individuals across 226 households with samples collected in March-June 2018 who underwent malaria laboratory testing, we exclude 19 (1.3%) individuals for missing results for at least one of the four diagnostic assays. These excluded individuals were not different from the included individuals. We have added the suggested information in the first two sentences of the results section (lines: 256–260):

> Among the 1,450 individuals across 226 households with samples collected in March-June 2018 who underwent malaria laboratory testing, we excluded 19 (1.3%) individuals for missing or failed results for at least one of the diagnostic assays— RDT, microscopy, PCR, and BBA. The 1,431 individuals included in this analysis were distributed across the study health areas as follows:

Reviewer 2, Comment 3: Please justify briefly why only HRP2 antigen positivity was utilized for the analysis, when more antigens were studied with BBA. Similarly, HRP2/pLDH RDTs were used, but only HRP2-ban positive results were reported (Table 1). Additional information could be interesting for the readers and added at least as supplementary material.

We report on the HRP2 antigen for BBA and the HRP2-band for RDT because the malaria burden is predominantly due to *P. falciparum* and to facilitate comparison for the detection of *P. falciparum* across the four assays (for example, to enable direct comparison to the HRP2 band on the RDT). We have included the distribution of other antigens tested with BBA and the pLDH-band for RDT in the S2 Table.

Reviewer 2, Comment 4: Were samples run in duplicates/triplicates for the multiplex bead-based immunoassay?

Due to the number of samples in this study, multiplex antigen detection samples were run in singlet. We have specified this in manuscript (lines: 169–171).

Though only HRP2 antigen positivity is utilized for analysis here, the standard BBA multiplex panel was utilized and performed in singleton for this sample set as described below.

Reviewer 2, Comment 5: In page 12, line 228 there is a typo: "stratifiedhealth areas" instead of "stratified health areas".

We have made the change.

Reviewer 2, Comment 6: In Table 1, I suggest to change "Age (years)" for "All age groups" in the row where overall numbers by health area are reported.

We have made the change.

Reviewer 2, Comment 7: In Table 1, authors refer to P. falciparum prevalence. However, any Plasmodium species visualized using microscopy was reported (as indicated in a footnote). Is information on species available? If yes, it could be used to restrict microscopy to those positive for P. falciparum. Are other species apart from P. falciparum important in Kinshasa Province?

Microscopy species determination is unfortunately not available. While other species are present in Kinshasa Province, they are found at low levels and usually occur as part of *P. falciparum* co-infection. We recently completed a separate analysis of non-falciparum infection in Kinshasa Province confirming this, but it has not yet completed peer review. However, our findings from a separate study of symptomatic malaria in three DRC provinces confirmed that <2% of all *Plasmodium* infections were caused by non-falciparum monoinfection (Parr et al. 2021 PMID: 33753817).

Parr JB, Kieto E, Phanzu F, Mansiangi P, Mwandagalirwa K, Mvuama N, et al. Analysis of false-negative rapid diagnostic tests for symptomatic malaria in the Democratic Republic of the Congo. Sci Rep. 2021;11: 6495. doi:10.1038/s41598-021-85913-z

Reviewer 2, Comment 8: In Table 1 footnote, letter a is repeated and letter e is missing.

We have made the change.

Reviewer 2, Comment 9: In Figure 2B, consider deleting the names within the figure and do a legend (as in Figure 2A) for simplification.

Thank you for this suggestion. We considered deleting the names with figure 2B, but elected to retain them to improve readability for readers who might struggle to discriminate differences in color.

Reviewer 2, Comment 10a: Diagnostic performance evaluation included sensitivity and specificity versus PCR/BBA/"alloyed gold standard", but other complementary measures could be used such as: positive predictive value, negative predictive value, positive likelihood ratio, negative likelihood ratio, diagnostic odds ratio, and area under the receiver operating characteristic curve.

Thanks for this suggestion. We have added S3 Table Positive and negative predictive values of malaria diagnostic methods versus PCR and BBA for the detection of *Plasmodium falciparum*, stratified by health area and malaria prevalence.

Reviewer 2, Comment 10b: Graphically, Venn diagrams could be included to visualize number of infections detected by each method and the overlapping. I suggest adding some of these measures/graphs in the section “Diagnostic performance for the detection of P. falciparum”.

We carefully considered this option but found Venn diagrams challenging to interpret with four (rather than three) categories to compare. We therefore used an alternative approach to visualize number of infections detected by each method and where they overlap (see Fig 3. Patterns of diagnostic assay results and distribution of health area [endemicity] for each pattern).

Reviewer 2, Comment 11: In Table 3, could %s be added (apart from the Ns) in the lower and upper 95% confidence limit?

We have incorporated the suggestion.

Reviewer 2, Comment 12: In introduction or discussion, new ultrasensitive malaria RDTs could be mentioned and put into context.

We have added a sentence to the discussion as suggested, noting that they have been developed but are not widely used.

Reviewer 3, Comment 1: Introduction: Please add malaria case data of three different endemic areas of Kinshasa province from the latest government report.

We have included the estimated total annual infections and provide detailed prevalence estimates as part of the modeling exercise.

Reviewer 3, Comment 2: Methods: Please provide number of household heads interviewed.

> We have added the suggested information in the first sentence of the results section (lines: 256–259):
>
> > Among the 1,450 individuals across 226 households with samples collected in March-June 2018 who underwent malaria laboratory testing, we excluded 19 (1.3%) individuals for missing or failed results for at least one of the diagnostic assays— RDT, microscopy, PCR, and BBA.

Reviewer 3, Comment 3: Line 124, it has been mentioned that "At the baseline visit of the second phase study…………………" but it has not been mentioned of endline in the manuscript.

> This paper is focused on diagnostic assay performance and included samples collected during the baseline/enrollment household visit (March-May 2018). Findings from the follow-up visits will be reported as part of future papers.

Reviewer 3, Comment 4: Table 1, malaria prevalence based on age distribution; the age categories are four but there are five rows of data. If the first row is of the total, the sum of age distribution data is not equal to total in Bu and Kimpoko. Please correct data. Similarly, self-reported fever in last seven days, the total of the two categories is not 1431. I am wondering why the number of people above 25 years in the study were less as compared to other age groups.

> Thank you for catching this. We have corrected the numbers in the tables and have added a footnote for missing data. However, note that the number of individuals ≥25 years old is higher than most other age categories (smallest is 15–24 year stratum).

Reviewer 3, Comment 5: In discussion, I suggest to add discussion on why there is variation in sensitivity and specificity of various diagnostic tests based on endemicity of malaria.

> It has been demonstrated previously that diagnostic performance varies by transmission intensity (see Ref #1) but that the relationship with prevalence is complex and not linear. However, in high prevalence regions, RDT positivity after clearance of parasitemia is expected to be common. We have included discussion of this in relation to our own findings in lines: 363–368).
